# Predictive Factors of the Standard Cross-linking Outcomes in Adult Keratoconus: One-Year Follow-Up

**DOI:** 10.1155/2017/4109208

**Published:** 2017-08-29

**Authors:** Amani E. Badawi, Waleed Ali Abou Samra, Ayman Abd El ghafar

**Affiliations:** Mansoura Ophthalmic Center, Faculty of Medicine, Mansoura University, Mansoura, Egypt

## Abstract

**Purpose:**

To evaluate the effects of preoperative presumed predictor factors on clinical and topographic outcomes in adult keratoconus (KC) 1 year after the standard corneal cross-linking (CXL).

**Design:**

Retrospective cohort study.

**Methods:**

The study included 84 KC patients (136 eyes) who were treated with conventional CXL. Postoperative best-corrected visual acuity (BCVA) and K max were considered the main predicted variables. The entire participants were divided into subgroups with cutoff values in accordance with the predictive variables. The predicted postoperative outcomes at one year were compared between the subgroups. Next, the predictive variables were analyzed by univariate and multivariate linear regression.

**Results:**

In respect to the BCVA, univariate analysis showed that the worse BCVA, the higher K max, and the relative thinner corneas were relatively good predictors of improvement, while multivariate evaluation revealed a strong interrelation with preoperative BCVA only. Regarding the postoperative flattening, univariate analysis found that the cone location and worse preoperative BCVA were the pronounced predictors, whereas the multivariate evaluation focused on the impact of the cone location only.

**Conclusions:**

The multivariate analysis disclosed a significant negative association between the baseline BCVA and postoperative BCVA and a positive relationship between the cone eccentricity and postoperative K max.

## 1. Introduction

Corneal collagen cross-linking (CXL) is one of the treatment modalities destined to reduce the disease progress of corneal ectasia and keratoconus (KC) [1]. The use of heat or light to augment the stromal collagen resistance has been started since the early 1990s [2]; therefore, the clinical and scientific implementation of KC management was originated by Wollensak et al. in 2003 [3].

Cross-linking procedure creates a photochemical reaction that aims to originate extra and new chemical chains in the anterior corneal stroma while minimizing harmful effects on the surrounding eye tissues [4]. This leads to increase the normal physical “anchors” inside the cornea with enhanced collagen cross-linking quality [5].

Numerous antecedent studies have proposed the efficiency of CXL in the optical and visual improvement of KC as well as in cessation of the disease progression with increasing the corneal biomechanical stabilization [6–8]. Indeed, the clinical efficiency of CXL may vary among the different patients. Thus, the ability to reliably forecast the postoperative outcomes before the procedure will assist the clinicians to reduce the undesirable consequences and to match the patients' anticipations [9].

There are a few guides on how the preoperative factors could impact on clinical outcomes of the CXL. Besides, diverse preoperative predictors of the standard CXL outcomes still need to be ascertained. In the current study, the main objective is to assess the effect of distinct preoperative demographic and topographic factors on the CXL results in adult KC after one year.

## 2. Patients and Methods

The study was designed as a retrospective cohort study. The collected data comprised 136 eyes of adult patients (above 18 years) with progressive KC grades 1–3 (based on Amsler-Krumeich grading). All recruited patients were treated by the standard epithelial-off CXL via the authors from 2013 till 2015. The surgical and study procedures were in accordance with the Declaration of Helsinki rules of researches on human beings. The study also was approved by the Mansoura University Faculty of Medicine (IRB: R1/17.06.71).

### 2.1. Surgical Technique

All calibrations of the “standard (epithelial-off) CXL” had been followed under the topical anaesthesia and totally sterile field. The corneal epithelium was removed mechanically within the central 8 mm. The isotonic riboflavin solution (0.1% in 20% dextran T500) was applied every 2-3 minutes for half an hour, following the complete stromal saturation; the cornea was exposed to the ultraviolet rays at the wavelength of 365 nm with a total surface irradiance of 3 mW/cm^2^. The riboflavin solution continued to be dripped every 2 minutes for another 30 minutes during the irradiation stage. Soft contact lenses were set on the corneas postoperatively and removed after the corneal epithelium was fully cured. Topical antibiotic drops were prescribed four times per day for one week, followed by topical corticosteroids for the subsequent 3 weeks; 4 times per day in the first week then the dose frequency was tapering gradually.

#### 2.1.1. Data Collection

The patients' data were collected retrospectively from the previous records. The selection of patients was based on particular standards, including those over the age of 18, maximum keratometry (K max) less than 58 D, the minimal corneal thickness at the thinnest location ≥ 400 microns, and a progressive disease. The preoperative progress of the disease was documented via corneal topography (at least two corneal topography within the former 6 months). Disease progression was documented by an increase of 1.0 diopter or more in K max reading and/or a decrease of the corneal thickness at the thinnest point of pachymetry by 10 *μ*m or more in the previous 6 months.

The family history of KC was defined as positive in those patients with first-degree relatives for KC. The corneal thickness less than 400 microns and patients who did not complete a follow-up for one year were excluded.

#### 2.1.2. Study Parameters

Postoperative contact lens best-corrected visual acuity (BCVA) and K max were considered the main predictive variables, while age, gender, positive family history of KC, baseline uncorrected visual acuity (UCVA), baseline BCVA, baseline K max, baseline thinnest pachymetry, and the cone location were the predictive variables. Firstly, the entire study participants were divided into subgroups with cutoff values in accordance with the defined predictive variables. The predicted postoperative outcomes at one year were compared between the subgroups. Next, the predictive variables were analyzed by linear regression for more accuracy.

## 3. Statistical Analysis

Statistical analysis was performed with SPSS program version 20 (SPSS, Chicago, IL). Descriptive statistical data were displayed as mean ± standard deviation (SD) for continuous data and as a number with a percentage for categorical data. The main study outcomes were changes in BCVA and changes in K max after one-year follow-up post-CXL. The paired sample *t*-test was conducted to analyze the changes in K max and BCVA (logMAR: logarithm of the minimum angle of resolution) between the baseline and at one year. The independent sample *t*-test was used in comparison between the subgroups regarding the parametric data, and the defined cutoff points were designated for dichotomizing the variables while the Mann–Whitney *U* test was used to analyze the nonparametric data. The *P* value of ≤0.05 was considered statistically significant. Then, all predicting factors were analyzed by univariate linear regression to determine the association between the study outcomes and the predictors. The normality of variables was checked by histograms. The *B* coefficients between the main predictive variables and the predictive variables were calculated; it represented how strongly the dependent variables (BCVA and K max) will change (positively or negatively) per each unit increase in the predictor. Next, to define the independent predictive factors, a multivariate linear regression was done; the predictive variables with the *P* value less than 0.20 in the univariate analysis model have been included again in the multivariate model.

## 4. Results

### 4.1. Demographic Characteristics

One hundred and thirty-six eyes of 84 patients were included, and the ages ranged between 18 and 33 years old (mean: 24.6 ± 1.32 years). 73.81% were females and 26.19% were males (Table 1).

### 4.2. Overall Outcomes at One Year

Table 2 shows the changes in mean different parameters between the baseline and at the end of one-year post-CXL and their *P* values in the whole study participants. The mean preoperative BCVA (logMAR) was remarkably improved (*P* = <0.001). By the end of 12 months follow-up, the K max values showed stabilization and maintained the baseline values in 50 eyes (36.76%) while K max improved and reduced (1–3.2 D) than the baseline values in 86 eyes (63.24%). No worsening or progression of the disease was recorded in any enrolled eyes.

### 4.3. Comparison between the Subgroups according to the Baseline Data

The entire study participants were divided into subgroups with cutoff values in accordance with age (≥30 years and less than 30 years), gender, family history of KC (positive and negative), preoperative K max (≥54 D and <54 D), preoperative BCVA (<0.3 and ≥0.3 logMAR), preoperative corneal thickness (<450 *μ* and ≥450 *μ*), and the cone locations (eccentric or centric) (Table 3).

#### 4.3.1. Age

Regarding the age, the overall patients were classified into 2 groups: patients' age < 30 years (*n* = 49 patients (58.3%)) showed a significant improvement in BCVA and the K max comparing to the baseline values (*P* = <0.001 and 0.022, resp.).

Patients' age ≥ 30 years (*n* = 35 patients (41.7%)) showed a significant improvement in both BCVA and K max (*P* = <0.001 and 0.023, resp.).

The BCVA showed a significant improvement in all age subgroups without a considerable difference (*P* = 0.152). Although the older patients showed more flatting in K max at one-year post-CXL, the comparison between the two subgroups regarding the K max changes was insignificant (*P* = 0.094).

#### 4.3.2. Gender and Family History

In male gender (*n*/22), the mean BCVA improved significantly from 0.37 ± 0.18 logMAR to 0.28 ± 0.14 logMAR (*P* = 0.035) and the mean K max changed from 49.44 ± 4.12 D to 48.18 ± 4.22 D (*P* = 0.02) at one-year post-CXL treatment. In female gender (*n*/62), there was a statistically significant improvement in both BCVA and K max (*P* = 0.02 and 0.01, resp.).

Comparing between the two subgroups showed insignificant differences in both BCVA and K max (*P* = 0.744 and 0.184, resp.). Concerning the family history, the difference between the subgroups (positive and negative family history of KC) was insignificant either in postoperative BCVA or postoperative topographic outcomes (*P* = 0.187 and 0.216, resp.).

#### 4.3.3. Preoperative BCVA

*(1) BCVA < 0.3 LogMAR (n* = 54* Eyes (39.7%))*. Those patients exhibited an insignificant improvement in both BCVA and K max. (*P* = 0.143 and 0.201, resp.). The mean BCVA changed from 0.21 ± 0.12 to 0.19 ± 0.01 and the K max changed from 48.32 ± 2.2 D to 47.85 ± 2.3 D.

*(2) BCVA ≥ 0.3 LogMAR (n* = 82* Eyes (60.3%))*. This subgroup showed a significant improvement in both BCVA and K max (*P* = <0.001 and 0.001, resp.). The mean BCVA changed from 0.55 ± 0.66 to 0.37 ± 0.33, and the K max changed from 50.81 ± 3.11 D to 48.75 ± 2.44 D.

The patients with worse baseline BCVA (≥0.3 logMAR) have obtained a higher benefit with respect to BCVA than those with better preoperative BCVA (*P* = <0.001). Similarly, the comparison between the subgroups concerning the changes in K max was significant (*P* = 0.004).

#### 4.3.4. Preoperative K max

The patients with preoperative K max ≥ 54 D (*n* = 63 eyes (46.3%)) showed a significant improvement in the postoperative BCVA and the mean K max (*P* ≤ 0.001 and 0.032, resp.). The mean BCVA changed from 0.65 ± 0.42 to 0.37 ± 0.33, and the K max changed from 56.48 ± 1.07 D to 55.82 ± 1.61 D.

In the patients with preoperative K max < 54 D (*n* = 73 eyes (53.7%)), the mean preoperative BCVA and K max showed significant improvement (*P* = <0.001 and 0.024, resp.). The mean BCVA changed from 0.41 ± 0.12 to 0.21 ± 0.11, and the K max changed from 46.77 ± 1.03 D to 46.11 ± 1.11 D.

When comparing the two subgroups, the higher K max seemed to be a good predictor for postoperative BCVA improvement, but the changes in K max did not show an obvious difference. *P* values were 0.020 and 0.122, respectively.

#### 4.3.5. Preoperative Thinnest Pachymetry

*(1) Pachymetry < 450 μ (n* = 62* Eyes (45.6%))*. The mean baseline thinnest location was 428.58 *μ* and changed to 411.53 *μ* (*P* = 0.001). The mean baseline BCVA in this subgroup changed significantly (*P* = 0.001), while the mean preoperative K max changed insignificantly (*P* = 0.085).

*(2) Pachymetry ≥ 450 μ (n* = 74* Eyes (54.4%))*. In those patients, the mean baseline pachymetry was 489.52 ± 5.32 *μ* and changed to 470.62 ± 6.15 *μ* (*P* = 0.001). The mean baseline BCVA in this subgroup changed significantly (*P* = <0.001), while the mean preoperative K max changed insignificantly (*P* = 0.845).

Comparison of the postoperative outcomes between the two subgroups showed that pachymetry less than 450 *μ* was a good predictor for postoperative BCVA improvement while it had an intelligible effect on the changes of K max. *P* values were 0.043 and 0.098, respectively.

#### 4.3.6. Cone Location

*(1) Eccentric Cone Location (n* = 37* Eyes (27.2%))*. Both the mean preoperative BCVA and the mean K max changed insignificantly (*P* = 0.061 and 0.724, resp.).

*(2) Central Cone Location (n* = 99* Eyes (72.8%))*. Both the mean preoperative BCVA and the mean K max changed significantly (*P* = 0.001 and 0.002, resp.).

Comparison of the postoperative outcomes between the two subgroups showed that the central cone was a significant predicting factor in flatting of the cornea but not on postoperative BCVA. *P* values were 0.001 and 0.187, respectively.

### 4.4. Univariate Analysis

Table 4 summarizes the univariate correlation between the supposed predictors and post-CXL BCVA and K max variables (dependent predictive variables). Neither the gender nor positive KC family history has an impact on any of treatment outcomes. Prominent predictors of the BCVA changes included BCVA, K max, and thinnest location, while the age factor, UCVA, and eccentricity were slight significant predictors of BCVA changes(*P* < 0.2). On the other hand, K max value change was significantly related to the changes in BCVA and eccentricity while UCVA, K max, and thinnest location were slight significant factors (*P* < 0.2). The significant values, *B* coefficients, and 95% CI are displayed in Table 4.

### 4.5. Multivariate Analysis

Concerning the BCVA outcome, the preoperative BCVA (logMar) was the only independent predictor (*P* value <0.001, *B* coefficient −0.800, and 95% CI 0.271–0.676). This means that the worse preoperative BCVA could be associated with more improvement in BCVA. Regarding the post-CXL corneal flattening, the cone eccentricity was the sole predictor (*P* value 0.0223, *B* coefficient 0.618, and 95% CI 0.097–1.170). This means that the more preoperative cone eccentricity, the less flattening of postoperative K max (Table 5).

### 4.6. Prediction Equation

To predict the postoperative BCVA (logMAR) at one-year post-CXL, the next equation was applied.

(1) $$\begin{array}{c} Y=0.622\;X+-0.040,\\ Y\left(dependent\;variable=postoperative\;BCVA\right),\\ X=\left(independent\;variable=preoperative\;BCVA\right). \end{array}$$

## 5. Discussion

The promising results of CXL in the management of either KC or corneal ectasia have encouraged the researchers to consider it as one of the substantial initial treatment procedures [10, 11]. Some published studies have addressed predictors of success for CXL; however, preoperative predictors of CXL efficiency were not entirely illustrated and there is still a necessity for further evaluation. Moreover, the discrepancy in the results has increased the incentive to understand more information on this theme.

In the current study, the gender factor and the family history did not have a significant influence on the treatment outcomes concerning both the BCVA and K max. Regarding the postoperative BCVA, the worse preoperative BCVA more than 0.3 logMAR, preoperative K max higher than 54 D, and preoperative pachymetry in the thinnest location less than 450 *μ* were good predictors for post-CXL improvement in BCVA. Whereas, the cone location had a negligible impact on postoperative BCVA.

With respect to the postoperative corneal flattening, worse preoperative BCVA (>0.3 logMAR) and the central cone seemed to be significant predictors of postoperative decrease in K max. While, patients age, the preoperative K max, and the preoperative corneal thickness showed insignificant impacts.

Currently, all age subgroups showed a significant improvement in BCVA and K max, and these findings were in agreement with Soeters et al. [12], Wisse et al. [9], and Godefrooij et al. [13]. Though the age was not a strong predictor of postoperative improvement of vision or corneal flattening, the most considerable results were in the patients above 30 years. This was consistent with another previous study by Toprak et al. [14]. They concluded that the patients older than 30 years had more postoperative corneal flattening compared with the results of younger patients.

On the other hand, Koller et al. [15] found that the patients aged more than 35 years were liable to more complications and had worse outcomes. Soeters et al. [12] and Godefrooij et al. [13] also reported better postoperative outcomes regarding BCVA in younger patients. They based these on the younger patients that had more frequent central cones compared to the adults [12], and the KC is more aggressive and more advanced in the pediatric group [16]. But this cannot be applied in the current study, as the progression was documented in all included eyes in different age groups as well as the study included adult patients only.

The worse preoperative BCVA (more than 0.3 logMAR) was a good predictor of both visual and topographic improvement. These results were consistent with many previous studies [9, 14] and inconsistent with Koller's team [17] who reported an insignificant impact of baseline BCVA on the corneal flattening after CXL treatment.

Regarding the K max, our findings were compatible with the results reported by Wisse et al. [9], while contrasting with other published studies which elucidated more prominent corneal flattening postoperatively in KC cases with higher preoperative K max [12, 17, 18].

Based on the current results, the baseline pachymetry seemed to be a robust predictor factor of BCVA improvement only and an insignificant impact on the corneal flattening. These results were opposite to the findings of Toprak et al. [14] who stated that the thinner cornea (<450 *μ*) exhibited more flattening postoperatively. In contrast, De Angelis et al.'s [19] results were in line with our results, as they reported better VA improvement in advanced KC stage (worse BCVA, higher K max, and thinner corneas).

Currently, the mean corneal thickness showed a significant reduction than the baseline values. Other studies reported the same results [20, 21] that this post-CXL thinning may due to anatomical and structural variations in the corneal collagen, keratocyte apoptosis, rearrangement of the collagen lamellae [22], or corneal ischaemia theory [23]. It is assumed that the changes in corneal thickness with the time is a sign of the disease progression of KC [24]. After CXL, corneal pachymetry becomes regularly thinner, so limiting corneal thickness role to document the early disease progression [25]. Therefore, it is hard to conclude the post-CXL thinning either due to the disease progression or CXL impacts.

Concerning the cone location, we found a different response between the central and the eccentric cones; the central cones seemed to respond well with more corneal flattening. Greenstein et al. [26] and Wisse et al. [9] recorded compatible results with a conclusion that the central cones had more postoperative corneal flattening comparing to the peripherally located cones. This finding could be interpreted by some facts. The CXL efficiency in the eccentric cones (3 mm apart from the center) decreases than that in the central cones as intended rays of CXL using currently available UV devices might not be homogeneous over the whole treating zone. The UV rays may disperse at the periphery with a less powerful and inconsistent beam in peripherally located cones. Therefore, the eccentric cones will exhibit less presumed clinical results [27]. The second fact is called “cosine effect.” This mathematical rule indicated that even with homogeneous distributed light energy, there was a relatively low treatment power in the peripheral cornea. In summary, the incidence angle of a ray with the corneal surface decreases towards the periphery, owing to the curvature of the cornea, and the light beam is falling over a wide corneal zone. Accordingly, the more peripheral cones may expose to less cross-linking power [27, 28].

In spite of the significant difference in topographic outputs between the central cones and the eccentric ones, the cone location did not display a significant impact on the BCVA. However, even with an insignificant difference between the two cone locations regarding BCVA improvement, we found that the central cone subgroup showed better improvement in BCVA than in the peripheral cones. This finding could be explained by the relationship between the visual acuity and the cone location. Whereas, the worst preoperative BCVA appeared to be closely related to the central cones [9].

The main aim of the study has been accomplished to reach the predicting factors of CXL success in adult KC. In respect to the BCVA, univariate analysis showed that the worse BCVA, the higher K max, and the relative thinner corneas were relatively good predictors of improvement, while multivariate evaluation revealed a strong interrelation with preoperative BCVA only. Regarding the postoperative flattening or topographic outcomes, univariate analysis found that the cone location and worse preoperative BCVA were the pronounced predictors, whereas the multivariate evaluation focused on the impact of the cone location only. These results are compatible with the prior studies in points and contrasted with them in other points. Therefore, there is a necessity for more studies to confirm what we have concluded. The strength of the present study is the relatively large sample size, considering each of the results obtained from both the univariate and multivariate analysis and the designing of a predictive estimating equation for the postoperative BCVA that can be applied later to make a relatively ideal decision. However, the retrospective design might be considered as a limitation of the study.

## 6. Conclusions

In conclusion, the current study proposed the impacts of some preoperative factors on the visual and topographic CXL outcomes. The multivariate analysis of the collected data from adult KC treatment disclosed a significant negative association between the baseline BCVA and postoperative BCVA which can be predicted and validated from the previously mentioned equation. The positive relationship was detected between the cone eccentricity and postoperative K max which could be used to foretell the post-CXL corneal flattening. These results could be worthy in clinical implementation for both ophthalmic surgeons and KC patients.

## Figures and Tables

**Table 1:** Demographic data of the patients.

Total number	136 eyes
Sex	22 males (26.19%)
	62 females (73.81%)
Age (years)	18–33 (24.6 ± 1.32) years
Laterality	32 patients (unilateral)
	52 patients (bilateral)
Family history (FH)	9 patients (positive FH)
	75 patients (negative FH)

**Table 2:** The changes in mean different parameters between the baseline and at the end of one-year post-CXL and their *P* values in the entire study.

Parameters in (136 eyes)	Baseline mean ± SD	One year mean ± SD	*P* value
UCVA (logMAR)	0.74 ± 0.10	0.52 ± .01	**<0.001** ^∗^
BCVA (logMAR)	0.38 ± 0.02	0.24 ± 0.12	**<0.001** ^∗^
K max (D)	49.16 ± 0.25	47.15 ± 0.25	**0.002** ^∗^
CCT (*μ*)	466.98 ± 6.21	455.47 ± 4.52	**<0.001** ^∗^
Thinnest location (*μ*)	454.76 ± 5.11	421.37 ± 3.24	**<0.001** ^∗^

CXL: crosslinking; SD: standard deviation; UCVA: uncorrected visual acuity; BCVA: best-corrected visual acuity; logMAR: logarithm of the minimum angle of resolution; K max: maximum K reading; CCT: central corneal thickness; test used: paired sample *t*-test; ^∗^*P* significant at the value <0.05.

**Table 3:** The changes in BCVA and K max at one year among the defined subgroups.

The defined subgroups	BCVA changes			K max changes		
	Mean difference	95% CI of the difference	*P* value	Mean difference	95% CI of the difference	*P* value
Age						
≥30	0.0521	−0.2244–0.07004	0.152	−1.0147	−1.56319–2.1514	0.094
<30						
Gender						
Male	0.0224	−0.4162–0.1089	0.744	1.47611	0.75234–2.70457	0.184
Female						
Preoperative BCVA						
≥0.3 logMAR	0.3192	0.2250–0.4134	**<0.001** ^∗^	2.7813	0.75532–4.8071	**0.004** ^∗^
<0.3 logMAR						
Preoperative K max						
≥54 D	0.1611	−0.1511–0.2734	**0.020** ^∗^	1.10143	0.52083–3.0843	0.122
<54 D						
Thinnest pachymetry						
≥450 *μ*	0.1214	−0.1527–0.1498	**0.043** ^∗^	−1.7817	−3.8985–0.33497	0.098
<450 *μ*						
Cone location						
Eccentric	0.0822	0.04124–0.2056	0.187	3.0263	1.6574–6.6032	**0.001** ^∗^
Central						

SD: standard deviation; BCVA: best-corrected visual acuity; K max: maximum K reading; CI: confidence interval; test used: independent *t*-test, ^∗^*P* significant at the value <0.05; P1: significance of BCVA changes between the subgroups; P2: significance of K max changes between the subgroups.

**Table 4:** Univariate linear regression of the baseline predictive factors and its significance on the treatment outcomes.

Baseline predictive factors	Changes in BCVA			Changes in K max		
	Standardized B coefficient	Significant *P* value	95% CI	Standardized B coefficient	Significant *P* value	95% CI
Age	0.0023	0.142	0.0025–0.125	−0.0142	0.695	−0.008–0.005
Gender (male predominance)	0.0115	0.621	0.752–1.874	0.0291	0.452	−0.582–1.285
Positive family history	0.008	0.854	−0.145–0.174	0.0983	0.345	0.184–1.114
UCVA (logMAR)	−0.917	0.140	0.350–0.449	0.931	0.162	1.450–2.374
BCVA (logMAR)	−0.945	**<0.001** ^∗^	0.505–0.615	−0.889	**<0.001** ^∗^	1.262–2.223
K max (D)	−0.816	**0.001** ^∗^	0.005–0.007	−1.000	0.081	0.981–0.991
Thinnest pachymetry (*μ*m)	0.792	**0.001** ^∗^	0.000–0.001	0.993	0.132	0.098–0.104
Eccentricity of the cone (mm)	0.794	0.101	0.152–0.236	0.946	**<0.001** ^∗^	1.716–2.149

BCVA: best-corrected visual acuity; UCVA: uncorrected visual acuity; logMAR: logarithm of minimal angle of resolution; K max: maximum keratometry; D: diopter; CI; confidence interval; *B* coefficient: the value which indicates how the dependent variable will vary per unit change in the predictive variable; test used: univariate linear regression test; ^∗^*P* Significant at the value <0.05.

**Table 5:** Multivariate linear regression of the baseline predictive factors and its significance on the treatment outcomes.

Baseline predictive factors	Changes in BCVA			Changes in K max		
	Standardized B coefficient	Significant *P* value	95% CI	Standardized B coefficient	Significant *P* value	95% CI
Age	0.018	0.642	−0.053–0.087	—	—	—
UCVA (logMAR)	0.342	0.113	−0.037–0.334	−0.002	0.856	−1.544–1.287
BCVA (logMAR)	−0.800	**<0.001** ^∗^	0.271–0.676	−0.004	0.643	−1.187–1.904
K max (D)	0.001	0.999	−0.007–0.007	0.794	0.070	0.931–1031
Thinnest pachymetry (*μ*m)	−0.280	0.517	−0.001–0.000	−0.014	0.555	−0.006–0.003
Eccentricity of the cone (mm)	0.069	0.631	−0.053–0.087	0.618	**0.0223** ^∗^	0.097–1.170

BCVA: best-corrected visual acuity; UCVA: uncorrected visual acuity; logMAR: logarithm of minimal angle of resolution; K max: maximum keratometry; D: diopter; CI; confidence interval; *B* coefficient: the value which indicates how the dependent variable will vary per unit change in the predictive variable; test used: multivariate linear regression test; ^∗^*P* significant at the value <0.05.
